# Prevalence of high-risk human papillomavirus infection and associated factors among women of reproductive age attending a rural teaching hospital in western Uganda

**DOI:** 10.1186/s12905-023-02342-y

**Published:** 2023-04-28

**Authors:** David Wol Nang, Happy Tukirinawe, Maxwell Okello, Bekson Tayebwa, Pius Theophilus, Franck Katembo Sikakulya, Yarine Fajardo, Adam Moyosore Afodun, Rogers Kajabwangu

**Affiliations:** 1 Department of Obstetrics and Gynecology, Kampala International University Teaching Hospital, Ishaka-Bushenyi, Uganda; 2 Department of Medical Laboratory Science, Kampala International University Teaching Hospital, Ishaka-Bushenyi, Uganda; 3 Department of Surgery, Kampala International University Teaching Hospital, Ishaka-Bushenyi, Uganda; 4 Department of Anatomy and Cell Biology, Faculty of Health Sciences, Busitema University, Tororo, Uganda

**Keywords:** High-risk human papillomavirus, Women of reproductive age, Genotype distribution

## Abstract

**Background:**

High-risk HPV is considered a major risk factor for the development of cervical cancer, the most common malignancy among women in Uganda. However, there is a paucity of updated epidemiological data on the extent of the burden and factors associated with hr-HPV infection among women of reproductive age. The aim of this study was to determine the prevalence and genotype distribution of hr-HPV and associated factors among women of reproductive age attending a rural teaching hospital in western Uganda.

**Methods:**

We conducted a cross-sectional study from April to June 2022. A total of 216 women of reproductive age attending the gynecological outpatient clinic were consecutively enrolled. Interviewer-administered questionnaires were used to collect participant characteristics, cervical specimens were collected by clinicians, and molecular HPV testing was performed using the Cepheid Xpert HPV DNA test. Descriptive statistics followed by binary logistic regression were conducted using SPSS version 22.

**Results:**

The prevalence of hr-HPV was 16.67%. Other hr-HPV types other than HPV 16 and 18 were predominant, with a prevalence of 10.6%; HPV 18/45 (2.31%), HPV 16 (0.46%), and 3.24% of the study participants had more than one hr-HPV genotype. On multivariate logistic regression, an HIV-positive status (aOR = 7.06, CI: 2.77–10.65, p = 0.007), having 3 or more sexual partners in life (aOR = 15.67, CI: 3.77–26.14, p = 0.008) and having an ongoing abnormal vaginal discharge (aOR = 5.37, CI: 2.51–11.49, p = 0.002) were found to be independently associated with hr-HPV infection.

**Conclusions and recommendations:**

The magnitude of hr-HPV is still high compared to the global prevalence. HIV-positive women and those in multiple sexual relationships should be prioritized in cervical cancer screening programs. The presence of abnormal vaginal discharge in gynecology clinics should prompt HPV testing.

## Background

Globally, human papillomavirus (HPV) is the most sexually transmitted virus and a major cause of morbidity and mortality [1]. Studies suggest that 75% of all sexually active people will become infected at some point during their lifetime [2]. Currently, over 120 HPV types have been identified, and persistent infection with hr-HPV genotypes is associated with the development of cervical intraepithelial lesions and cancer [3].

Cervical cancer is the world’s fourth most prevalent cancer among women and accounts for 6.5% of all female cancers [4]. Between 2018 and 2030, the annual number of new cases of cervical cancer is expected to rise from 570 000 to 700 000, with 311 000 to 400 000 fatalities [4]. To eliminate cervical cancer by 2030, 70% of women must be screened with a high-performance test by the age of 35 and then again by the age of 45 [5].

Uganda has a population of 12.3 million women of reproductive age who are at risk of developing cervical cancer. Cervical cancer ranks as the most frequent cancer among women in Uganda, and approximately 21% of women in the general population are estimated to harbor cervical HPV-16/18 infection at a given time [6]. Approximately 70% of invasive cervical cancers are attributed to HPVs 16 or 18 [6].

Currently, the common methods used for cervical cancer screening in Uganda depend on cytological and histological methods. It has, however, been shown that both cytological and histological findings are not good indicators for the presence of HPV since most women with an HPV infection do not present with microscopic abnormalities of the cervical cells [7]. The World Health Organization currently recommends using HPV DNA detection as the primary screening test rather than visual inspection with acetic acid (VIA) or cytology in screening and treatment approaches among both the general population of women and women living with HIV [8]. Invasive cervical cancer can be prevented by combining screening efforts with prompt treatment of all screen positives for HPV infection. This has proven to be the most effective intervention in narrowing the current prevention gap to date [9].

A previous study conducted in the Bushenyi district in the rural western region of Uganda found a significant prevalence (17.2%) of hr-HPV among women living in rural areas [10]. Both the prevalence and factors associated with hr-HPV may have changed over time as a result of rapid expansion of the population and urbanization, which have occurred throughout the time in the Bushenyi district. This calls for the need to generate updated epidemiological data on the extent of the burden and factors associated with hr-HPV among women of reproductive age attending a major rural teaching hospital in western Uganda.

## Methods

### Study design, area and population

We conducted a health facility-based cross-sectional, descriptive and analytical study at Kampala International University Teaching Hospital (KIU-TH), which is located in Ishaka-Bushenyi municipality, approximately 319.7 km from Kampala city on the Kampala-Mbarara-Kasese highway, 60 km west of Mbarara. We targeted all women who attended the obstetrics and gynecological outpatient clinic, and our study population was all women of reproductive age who met the eligibility criteria. The study was carried out over a period of three months (April to June 2022).

### Inclusion criteria

Women of reproductive age (15 to 49 years), including pregnant mothers who attended the obstetrics and gynaecology outpatient department of KIU-TH, were included in this study.

### Exclusion criteria

Women who had undergone total hysterectomy, women with obvious cervical lesions or masses that bleed on contact, and those who were mentally or physically unable to undergo a pelvic examination were excluded from the study.

### Sampling size determination

The sample size was calculated using the Kish Leslie formula (1965):

$$n=\frac{{z}^{2}p\left(1-p\right)}{{e}^{2}}$$

n: Estimated minimum sample size required.

p: Proportion of a characteristic in a sample.

e: The acceptable margin of error set at 5%.

z: 1.96 (for 95% confidence interval).

In a population-based cross-sectional study conducted by Asiimwe et al. (2008) among residents of Sheema County in Bushenyi District, the prevalence of high-risk HPV was 17.2%.

$$n=\frac{{\left(1.96\right)}^{2}\times 0.17\times (1-0.17)}{{\left(0.05\right)}^{2}}=216$$

### Pilot study and sampling technique

Women who met the eligibility criteria were enrolled in a pilot study with an initial (n of N) number of participants derived from Cochran’s formula to eliminate prejudice and determine the feasibility of this study. Patients were recruited consecutively from obstetrics and gynecology outpatient clinics until the desired sample size was achieved. A unique study number was assigned to each participant to avoid duplication. The methodology of our screening was an open trajectory to avoid prejudice or bias. There was no patient reward system or inducement, and participation was purely voluntary.

### Diagnosis of high-risk HPV

After obtaining consent from the study participants, the interviews, physical examination and sample collection were performed in a separate room from other clients to maintain privacy and confidentiality. Sociodemographic, medical, obstetrical and gynecologic factors of study participants were captured using a pretested and standardized questionnaire written in English and the local language (Runyankole). Cervical specimens for HPV testing were collected by inserting a sterile vaginal speculum into the vagina. The specimens were collected from the ectocervical and endocervical regions using the Cervexbrush25, and samples were deposited into Preservcyt50 transport medium from each participant. The specimens were transported at room temperature to the KIU-TH biochemistry laboratory for the detection of hr-HPV by a trained laboratory technician (with a Bachelor of Science in Medical laboratory science qualification).

### HPV genotyping

HPV DNA genotyping was carried out using Cepheid’s Xpert HPV test (GXHPV-CE-10) using a GeneXpert machine from Cepheid (Sunnyvale, CA, U.S. A), located at the KIU-TH laboratory. For cervical specimens in Presevercyt, the Xpert HPV test has a sensitivity of 90.0% and 94.5% and a specificity of 43.5% and 41.3% relative to ≥ CIN2 and ≥ CIN3 disease status, respectively (www.cepheidinternational.com).

Thin prep vials containing the samples were first pretreated with 3 ml of concentrated glacial acetic acid (GAA) to lyse the cells. The lysed cells were gently vortexed at half speed continuously for approximately five seconds. After vortexing, 2 ml of the sample was loaded using a pipette into the sample chamber of the Xpert HPV Assay cartridge, and the cartridge lid was closed. The Xpert HPV Assay Definition File (ADF) was then imported into Gene Xpert software, which launches automatically. The Xpert HPV Assay (which is a multiplex Real-time PCR) includes reagents for the simultaneous detection of 13 h-HPV types (HPV16, 18, 31, 33, 35, 39, 45, 51, 52, 56, 58, 59, and 68) and one possible hr-HPV type (HPV66) in 1 h, a human reference gene (hydroxymethylbilane synthase [HMBS]) or specimen adequacy control, and an internal probe check coxbntrol (PCC). The 14 targeted HPV types were detected in five fluorescent channels: (1) HPV16; (2) HPV18 and HPV 45 (HPV18/45); (3) HPV31, 33, 35, 52, and 58 (HPV31/33/35/52/58); (4) HPV51 and HPV59 (HPV51/59); and (5) HPV39, 56, 66, and 68 (HPV39/56/66/68). The specimen adequacy control, HMBS, was detected in a sixth fluorescent channel. The PCC verifies reagent rehydration, PCR tube filling in the cartridge, probe integrity, and dye stability. In total, the assay uses six fluorescent channels for the detection of individual types of HPV, groups of HPVs, and the human reference gene. Each fluorescent channel contained its own cut-off parameters for target detection/validity. Assay results were reported as HPV16 and HPV18/45 specifically as “positive” or “negative.” Collectively, the other high-risk types of HPV detected by the assay were reported in a pool.

### Data analysis

The dataset was developed using Microsoft Excel version 10 and was imported into SPSS version 22 for analysis. The prevalence of high-risk human papillomavirus was calculated as a fraction of participants with high-risk human papillomavirus against all participants enrolled in the study and expressed as a frequency and percentage. The results were then presented in the text using a pie chart. The factors associated with high-risk human papillomavirus were analysed using binary logistic regression (bivariate and multivariate). Both unadjusted (crude) odds ratios with their corresponding 80% confidence intervals (CIs) and adjusted ORs with their corresponding 95% CIs were reported. Variables that were statistically significant at the bivariate level or had a p ≤ 0.2 were moved to the multivariate level. A p value ≤ 0.05 was considered statistically significant at the multivariable level. The results are then presented in table form.

## Results

### Characteristics of the study population

The mean age of the 216 participants we enrolled was 30 ± 7.2 **(**Table 1**).** The majority of these were between the ages of 25 and 29 (30.1%), from rural areas (69.9%), married (69.9%), peasants (50.5%), earning less than 200,000/= per month (79.6%), HIV negative (80.1%), and never taking alcohol in the past year (77.8%).

The mean age at menarche was 14 ± 1.6, at coitarche (18 ± 2.6) **(**Table 1**)**, and at first childbirth (18 ± 7.5). The majority of participants (94.9%) were not pregnant, while 79.6% used contraceptives and 78% did not use condoms. The majority had one sexual partner in the previous year (77.3%), and 50.5% had an STI in that time period **(**Table 1**)**, while the majority had ongoing abnormal vaginal discharge (64.4%).

### Prevalence of high-risk HPV infection

**Fig. 1:**
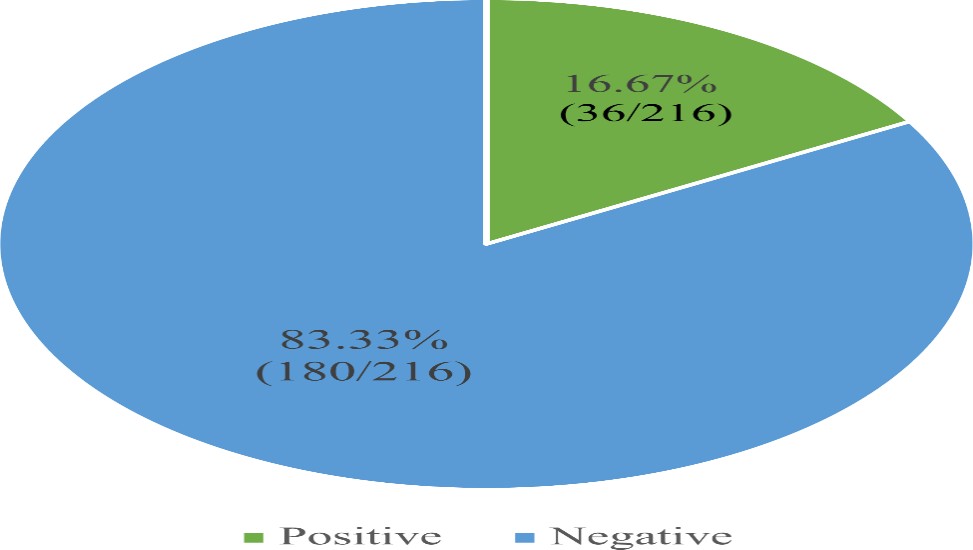
Pie chart showing the prevalence of hr-HPV infection

Detection of hr-HPV was performed on 216 study participants; 6 of the tests were initially invalid, but these invalid tests were repeated, and the corresponding result was recorded. A total of 36/216 of the study participants tested positive for hr-HPV; hence, the prevalence of hr-HPV among women of reproductive age attending KIU-TH was 16.67% **(**Fig. 1**).**

**Fig. 2:**
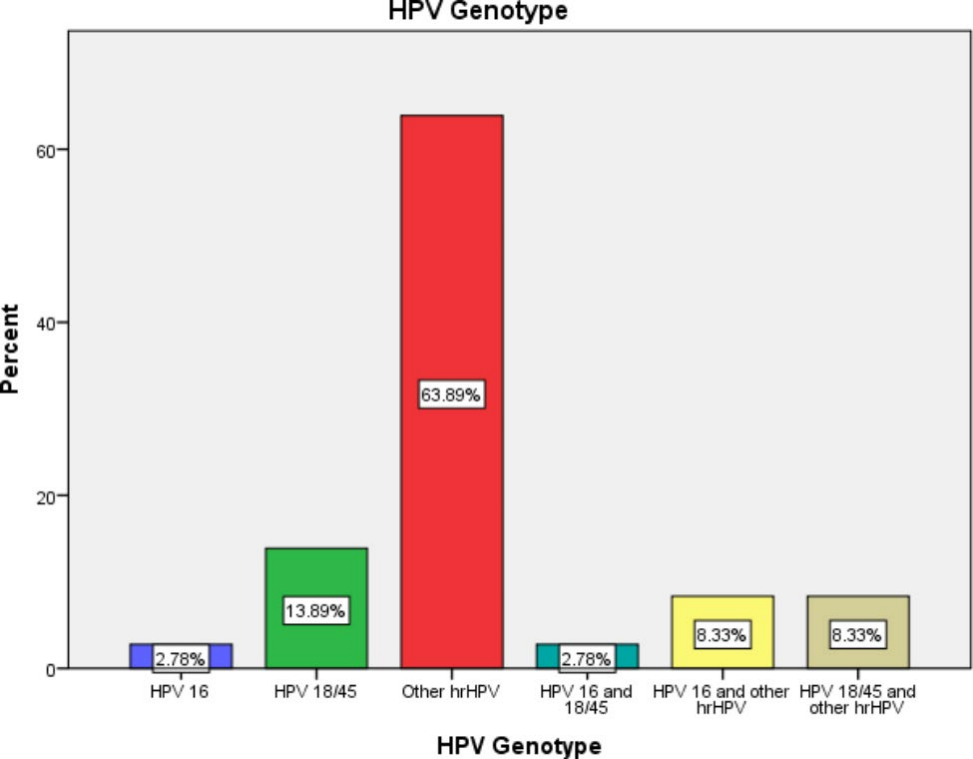
A bar graph showing the genotype distribution of hr-HPV; HPV 16 = 2.78%, HPV 18/45 = 13.89%, other hrHPV = 63.89%, HPV 16 & 18/45 = 2.78%, HPV 16 & other hr HPV = 8.33%, HPV 18/45 & other hr HPV = 8.33%.

The most common genotypes were the other high-risk HPV genotypes (31, 33, 35, 39, 51, 52, 56, 58, 59, 66, and 68), with a prevalence of 10.6% and accounting for 63.89% of all hr-HPV-positive participants. HPV16 had a prevalence of 0.46% and accounted for 2.78% of all hr-HPV-positive participants. HPV 18/45 had a prevalence of 2.31% and accounted for 13.89% of all hr-HPV-positive patients. A total of 3.24% of the study participants had more than one genotype, accounting for 19.4% of all positive participants **(**Fig. 2**).**

### Factors associated with hr-HPV infection

In bivariate analysis **(**Table 1**and** Table 2**)**, the variables that showed an association with hr-HPV infection (P value ≤ 0.2) were; residence, marital status, educational level, occupation, HIV status, alcohol intake in the past year, menarche, coitarche, contraceptive use, number of sexual partners in the past year, number of sexual partners for life, having an STI in the past year and the presence of an ongoing abnormal vaginal discharge. On multivariate regression **(**Table 3***)***, the independent significant factors associated with hr-HPV infection (P value ≤ 0.05) included; HIV status, the number of sexual partners in life time, and the presence of an ongoing abnormal vaginal discharge. Women with HIV were 7 times (**aOR = 7.06, CI: 2.77–10.65, p = 0.007**) more likely to test positive for hr-HPV than those who did not. In comparison to women who had one sexual partner in life, those who had three or more were 16 **times (aOR = 15.67, CI: 3.77–26.14, p = 0.008)** more likely to test positive for hr-HPV. Women with an ongoing abnormal vaginal discharge had a higher risk of testing positive for hr-HPV (5 times) than those who did not (**aOR = 5.37, CI: 2.51–11.49, p = 0.002**).

**Table 1:** The results of bivariate analysis for sociodemographic and medical factors associated with hr-HPV infection among women of reproductive age attending KIU-TH.

Variable	Number n (%) (N = 216)	High risk HPV status n (%)		cOR (95% CI)	P value
		**Negative** (n = 180)	**Positive**(n = 36)		
**Age**					
15–19	4(1.9)	3(75.3)	1(25.0)	3.33(0.45–24.61)	0.440
20–24	46(21.3)	40(87.0)	6(13.0)	1.50(0.35–6.44)	0.721
25–29	65(30.1)	57(87.7)	8(12.3)	1.40(0.34–5.86)	0.761
30–34	41(19.0)	33(80.5)	8(19.5)	2.42(0.58–10.19)	0.429
35–39	33(15.3)	28(84.8)	5(15.2)	1.79(0.41–7.85)	0.616
40–44	16(7.4)	9(56.3)	7(43.8)	2.33(0.20-27.57)	0.501
45–49	11(5.1)	10(90.9)	1(9.1)	Ref	
**Residence**					
Urban	67(31.0)	60(89.6)	7(10.4)	Ref	
Rural	149(69.0)	120(80.5)	29((19.5)	2.071(1.16–36.87)	0.105*
**Marital status**					
Married	151(69.9)	128(84.8)	23(15.2)	Ref	
Single	44(20.4)	38(86.4)	6(13.6)	0.88(0.47–1.66)	0.794
Divorced	14(6.5)	8(57.1)	6(42.9)	4.17(1.97–8.84)	0.015*
Widow	7(3.2)	6(85.7)	1(14.3)	0.93(0.23–3.82)	0.946
**Education level**					
Tertiary	51(23.6)	48(94.1)	3(5.9)	Ref	
Secondary	63(29.2)	60(95.2)	3(4.8)	0.80(0.27–2.35)	0.790
Primary	78(36.1)	56((71.8)	22(28.2)	6.29(2.75–14.39)	0.004*
No formal education	24(11.1)	16(66.7)	8(33.3)	8.00(3.12–20.55)	0.005*
**Formal employment**					
Private sector	34(15.7)	30(88.2)	4(11.8)	Ref	
Public sector	4(1.9)	4(100)			
Business	45(20.8)	40(88.9)	5(11.1)	0.94(0.38–2.34)	0.928
Peasant	109(50.5)	84(77.1)	25(22.9)	2.23(1.06–4.69)	0.165*
Student	24(11.1)	22(91.7)	2(8.3)	0.68(0.21–2.19)	0.674
**Monthly income (Ugx)**					
> 1,000,000	2(0.9)	2(100)			
500,000–1,000,000	16(7.4)	15(93.7)	1(6.3)	Ref	
200,000–499,999	26(12.0)	22(84.6)	4(15.4)	2.73(0.61–12.17)	0.390
< 200,000	172(79.6)	141(82)	31(18)	3.30(0.86–12.69)	0.257
**HIV status**					
Negative	173(80.1)	158(91.3)	15(8.7)	Ref	
Positive	43(19.9)	22(51.2)	21(48.8)	10.06(4.53–22.35)	< 0.001*
**Alcohol intake past year**					
No	168(77.8)	148(88.1)	20(11.9)	Ref	
Yes	48(22.2)	32(66.7)	16(33.3)	3.70(1.73–7.92)	< 0.001*

*P ≤ 0.2; ugx = Uganda shillings; cOR: crude odds ratio

**Table 2:** The results of bivariate analysis for gynecological and obstetrical factors associated with hr-HPV infection among women of reproductive age attending KIU-TH.

Variable	Number n (%) (N = 216)	High risk HPV status n (%)		cOR (95% CI)	P value
		**Negative** (n = 180)	**Positive** (n = 36)		
**Menarche**					
≤ 12 years	26(12.0)	17(65.4)	9(34.6)	3.20(1.29–5.78)	0.012*
> 12 years	190(88.0)	163(85.8)	27(14.2)	Ref	
**Coitarche**					
≤ 15 years	28(13.0)	20(71.4)	8(28.6)	2.29(1.26–4.15)	0.076*
> 15 years	188(87.0)	160(85.1)	28(14.9)	Ref	
**Currently pregnant**					
No	205(94.9)	171(83.4)	34(16.6)	Ref	
Yes	11(5.1)	9(81.8)	2(18.2)	1.12(0.23–5.40)	0.890
**Parity**					
0	34(15.7)	30(88.2)	4(11.8)	Ref	
1–3	128(59.3)	110(85.9)	18(14.1)	0.33(0.05–2.33)	0.268
4–6	47(21.8)	35(74.5)	12(25.5)	0.41(0.07–2.27)	0.307
> 6	7(3.2)	5(71.4)	2(28.6)	0.86(0.15–5.01)	0.864
**Age at 1st term birth**					
Nulliparous	27(12.5)	24(88.9)	3(11.1)	Ref	
< 17 years	15(6.9)	12(80.0)	3(20.0)	2.00(0.64–6.26)	0.436
17–20 years	93(93)	71(76.3)	22(23.7)	2.48(1.06–5.77)	0.368
> 20 years	81(37.5)	73(90.1)	8(9.9)	0.88(0.35–2.20)	0.854
**Contraceptive use**					
No	44(20.4)	41(93.2)	3(6.8)	Ref	
Yes	172(79.6)	139(80.8)	33(19.2)	3.23(0.95–11.13)	0.061*
**Partner circumcised**					
No	113(52.8)	89(78.8)	24(21.2)	0.49(0.23–1.04)	0.062*
Yes	103(47.7)	91(88.3)	12(11.7)	Ref	
**Condom use**					
No	169(78.2)	141(83.4)	28(16.6)	1.033(0.44–2.45)	0.941
Yes	47(21.8)	39(83.0)	8(17.0)	Ref	
**Number of sexual partners past year**					
0	4(1.9)	4(100)			
1	167(77.3)	147(88)	20(12.0)	Ref	
2	40(18.5)	27(67.5)	13(32.5)	3.54(1.57–7.95)	0.002*
≥ 3	5(2.3)	2(40.0)	3(60.0)	11.04(3.29–36.94)	0.011*
**Number of sexual partners in life**					
1	96(44.4)	92(95.8)	4(4.2)	Ref	
2	57(26.4)	52(91.2)	5(8.8)	2.21(0.57–8.59)	0.252
≥ 3	63(29.2)	36(57.1)	27(42.9)	17.25(5.64–52.79)	< 0.001*
**STIs in past year**					
No	107(49.5)	103(96.3)	4(3.7)	Ref	
Yes	109(50.5)	77(70.6)	32(29.4)	10.70(3.63–31.69)	< 0.001*
**Ongoing abnormal vaginal discharge**					
No	139(64.4)	133(95.7)	6(4.3)	Ref	
Yes	77(35.6)	47(61.0)	30(39.0)	14.15(5.54–36.13)	< 0.001*

*P ≤ 0.2; STIs; sexually transmitted infections. cOR: crude odds ratio

**Table 3:** The results of multivariate analysis for factors associated with hr-HPV infection among women of reproductive age attending KIU-TH.

Variable	Number n (%) (N = 216)	High risk HPV status n (%)		cOR (95% Cl)	P value	aOR (95% Cl)	P Value
		Negative (n = 180)	Positive (n = 36)				
Residence							
Urban	67(31.0)	60(89.6)	7(10.4)	Ref		Ref	
Rural	149(69.0)	120(80.5)	29(19.5)	2.07(1.16–36.87)	**0.105**	27(0.05–14.92)	0.302
**HIV status**							
Negative	173(80.1)	158(91.3)	15(8.7)	Ref		Ref	
positive	43(19.9)	22(51.2)	21(48.8)	10.06(4.53–22.35)	**< 0.001**	7.06(2.77–10.65)	0.007**
**Alcohol intake**							
No	168(77.8)	148(88.1)	20(11.9)	Ref		Ref	
Yes	48(22.2)	32(66.7)	16(33.3)	3.70(1.73–7.92)	**< 0.001**	6.48(0.60–6.98)	0.123
**Menarche**							
≤ 12 years	26(12.0)	17(65.4)	9(34.6)	3.20(1.29–5.78)	**0.012**	3.96(0.09–1.48)	0.508
> 12 years	190(88.0)	163(85.8)	27(14.2)	Ref		Ref	
**Coitarche**							
≤ 15 years	28(13.0)	20(71.4)	8(28.6)	2.29(1.26–4.15)	**0.076**	0.84(0.04–1.69)	0.910
> 15 years	188(87.0)	160(85.1)	28(14.9)	Ref		Ref	
**Contraceptive use**							
No	44(20.4)	41(93.2)	3(6.8)	Ref		Ref	
Yes	172(79.6)	139(80.8)	33(19.2)	3.23(0.95–11.13)	**0.061**	0.24(0.01–8.91)	0.437
**Number of sexual partners past year**							
0	4(1.9)	4(100)					
1	167(77.3)	147(88)	20(12.0)	Ref		Ref	
2	40(18.5)	27(67.5)	13(32.5)	3.54(1.57–7.95)	**0.002**	0.45(0.01–15.02)	0.652
≥ 3	5(2.3)	2(40.0)	3(60.0)	11.03(3.29–36.94)	**0.011**	4.04(0.61–12.61)	0.063
**Number of sexual partners in life**							
1	96(44.4)	92(95.8)	4(4.2)	Ref		Ref	
2	57(26.4)	52(91.2)	5(8.8)	2.21(0.57–8.59)	0.252	2.50(0.07–9.44)	0.652
≥ 3	63(29.2)	36(57.1)	27(42.9)	17.25(5.64–52.79)	**< 0.001**	15.7(3.77–26.14)	0.008**
**STIs in past year**							
No	107(49.5)	103(96.3)	4(3.7)	Ref		Ref	
Yes	109(50.5)	77(70.6)	32(29.4)	10.70(3.63–31.69)	**< 0.001**	1.61(0.09–3.02)	0.748
**Ongoing abnormal vaginal discharge**							
No	139(64.4)	133(95.7)	6(4.3)	Ref		Ref	
Yes	77(35.6)	47(61.0)	30(39.0)	14.15(5.54–36.13)	**< 0.001**	5.37(2.51–11.49)	0.002**

**P ≤ 0.05; STIs: sexually transmitted infections; cOR: crude odds ratio; aOR; adjusted odds ratio

## Discussion

The aim of this study was to determine the prevalence of high-risk human papillomavirus infection and associated factors among women of reproductive age attending Kampala International University-Teaching Hospital.

The overall prevalence of hr-HPV in this study was 16.67%. Our results are in line with previous studies in Uganda and other African countries where the prevalence ranged between 17% and 25% [10–13]. The high prevalence in these countries is a negative reflection of HPV vaccination programs, which have been largely ineffective due to poor infrastructure and inadequate funding [14]. Developed countries such as Australia, which have highly effective HPV vaccination programs, have reduced HPV infection rates to as low as 2.3% [15]. For cervical cancer to be eliminated, there is a need to strengthen vaccination programs to cover at least 90% of girls below 15 years of age, as recommended by the World Health Organization [16]. This is critical, especially in Sub-Saharan African countries such as Uganda, where most deaths from cervical cancer occur.

In this study, the most common genotypes were the other high-risk HPV genotypes (31, 33, 35, 39, 51, 52, 56, 58, 59, 66, and 68), followed by HPV 18/45 and HPV 16. However, a higher number among those who tested positive for hr-HPV had more than one genotype. These findings are in agreement with studies that employed similar molecular techniques of hr-HPV DNA detection, such as findings found by [17] in Swaziland, where the majority (45.3%) of the participants had other high-risk HPV, 12.4% had HPV 16 and 13.8% had HPV 18/45, and in India, where the majority (33.3%) had other high-risk HPV and 16.67% had HPV 18/45 [18]. Whereas most cervical cancer cases have been previously attributed to HPV 16 and 18, there is now growing evidence on the importance of other high-risk HPV serotypes in cervical cancer causation. As more evidence is generated, there will be a need to review the vaccination strategies to avail the nonavalent vaccine in all settings.

In our study, being HIV positive significantly increased the risk of hr-HPV infection. This increase in risk has been documented in a number of previous studies [6, 13, 19]. HIV infection causes immune suppression, increasing the likelihood of HPV acquisition and delayed clearance [20]. Having 3 or more sexual partners in life was also associated with an increase in the risk of HPV infection, as was found by Nascimento and colleagues in a Brazilian study and Okunade in a Nigerian study [21, 22]. These findings imply that cervical cancer prevention programs in resource-limited settings should prioritize meagre HPV testing resources to HIV-positive clients and those who are likely to have multiple sexual partners, such as sex workers.

Our findings on relatively high rates of HPV infection among women with abnormal vaginal discharge are in line with those found in Spain [23]. The loss of protecting microorganisms and other changes brought on by vaginal infections could make it easier for other sexually transmitted infections, such as HPV, to be easily acquired and persist. Clinicians managing patients should consider performing HPV screening on them.

### Strengths and limitations

This is the second study in our region to report hr-HPV prevalence among women of reproductive age. Our findings update epidemiological knowledge about the burden, genotype distribution, and risk factors associated with hr-HPV infection in an understudied rural population in western Uganda.

Our study, however, was a hospital-based study, and we believe that in order to obtain more precise data on distribution and prevalence rates in this geographical region, we need to expand this study to the community level, including both the rural and urban sectors. In addition, the Xpert HPV DNA test reports HPV16, 18/45, and 11 other high-risk types in a pool. Therefore, we were not able to separately determine the prevalence of the other 11 types of hr-HPV. A more advanced kit of ELISA HIV Ab-Ab/In-Tec 136 or HIV-1 p24 Antibody (FITC) would be recommended in further research depending on the global availability of its marketing patents and trademark.

## Conclusion and recommendations

Our findings indicate that hr-HPV prevalence in our setting is still high compared to the global prevalence. Other than types 16 and 18/45, the most prevalent genotypes in our population are the other hr-HPV, but coinfections are also frequent.

The factors independently associated with an increased risk of hr-HPV infection were HIV-positive status, having 3 or more sexual partners in life, and having an ongoing abnormal vaginal discharge.

HIV-positive women and those in multiple sexual relationships should be prioritized in cervical cancer screening programs. The presence of abnormal vaginal discharge in gynecology clinics should prompt clinicians to perform HPV testing on affected women.

## Data Availability

materials: The datasets generated and/or analysed during the current study are not publicly available due to the limitations of ethical approval involving the patient data and anonymity but are available from the corresponding author upon reasonable requests.

## Abbreviations

Hr- HPV: High-risk human papillomavirus
KIU-TH: Kampala International University Teaching Hospital.
KIU-WC: Kampala International University - Western campus.
